# An exploratory study of the relationship between parental attitudes and behaviour and young people's consumption of alcohol

**DOI:** 10.1186/1747-597X-5-6

**Published:** 2010-04-22

**Authors:** Graham F Moore, Heather Rothwell, Jeremy Segrott

**Affiliations:** 1 Cardiff Institute of Society and Health, Cardiff School of Social Sciences, Cardiff University, 1-3 Museum Place, Cardiff, CF10 3BD UK

## Abstract

**Background:**

Concern is growing regarding frequent and excessive misuse of alcohol by young people. The average age at which young people in Europe start to drink is twelve and a half, and during the last decade, the quantity of alcohol consumed by younger adolescents in the UK has increased. Families are known to play an important role in shaping young people's alcohol misuse, although family risk and protective factors associated with misuse in a UK context are in need of further investigation.

**Methods:**

The study used a cross-sectional design, involving secondary analyses of self-completion questionnaire responses from 6,628 secondary school children (i.e. aged 11-16 years), from 12 schools within an urban location in Wales. Items relating to family functioning and perceived parental attitudes were first subjected to factor analysis. Associations of family closeness and conflict, parental monitoring and attitudes and family history of substance misuse with children's self reported alcohol consumption were examined using logistic regression analyses.

**Results:**

Approximately three quarters of respondents reported having tried alcohol, most of whom had first tried alcohol aged 12 or under. Parental monitoring and family closeness were positively correlated with one another and were both associated with significantly lower levels of drinking behaviours. Family violence and conflict, more liberal parental attitudes towards substance use and towards alcohol and petty crime, and family history of substance misuse were positively correlated with one another and with higher levels of drinking behaviours. Parental monitoring was identified as the family functioning factor most consistently associated with drinking behaviour in multivariate analyses.

**Conclusions:**

Significant relationships were found between young people's drinking behaviours and perceptions of risk and protective factors in the family environment. Parental monitoring was strongly associated with family closeness and appeared to form one part of a parenting style of more general communication and regulation of children's behaviour. Findings support the need for alcohol misuse prevention interventions which address risk and protective factors within the family setting. Timing of such prevention work should be related both to the development of family relationships and the age at which young people begin drinking alcohol.

## Background

The risk of alcohol-related harm in adult life is inversely related to the age at which individuals begin to drink alcohol [1-3]. During recent years concern has grown regarding frequent and excessive misuse of alcohol by young people [4,5]. The average age at which young people in Europe start to drink is twelve and a half [6] and during the last decade, the quantity of alcohol consumed by younger adolescents in the UK has increased [7]. Of the forty countries taking part in the 2005/2006 European Health Behaviour in School Children (HBSC) survey, Wales had the highest proportion of 13 year-olds (26% of girls and 27% of boys) who had been drunk at least twice. Wales was also in the top 13 countries for weekly drinking at ages 11 (4% of girls and 7% of boys), 13 (20% of girls and 23% of boys) and 15 (38% of girls and 42% of boys), and for reporting of having first become drunk at age 13 or younger (21% of girls and 25% of boys aged 15) [8]. Prevention of alcohol-related harm must therefore address influences on children which lead them to begin drinking early in life.

In the UK, measures to prevent alcohol misuse amongst children usually involve school-based education about alcohol and substance misuse [9-11]. There is little evidence however that programmes based solely upon classroom-based learning have been effective in changing behaviour [12]. Thus recent attention has focused on understanding family influences on young people's drinking. Family relationships and interactions are central influences on children's behaviour [13-15], and have been shown, mostly in US based studies, to influence the timing of young people's alcohol use [16]. Parental involvement and intervention at primary-school age, when family influences are relatively strong, have been identified as important in increasing the effectiveness of programmes to prevent alcohol misuse [17-21]. However, despite high levels of alcohol misuse among Welsh schoolchildren, few studies have explored family risk and protective factors for alcohol consumption in Wales or the UK.

One theoretical model for understanding potential influences of family functioning upon children's behaviours is the Social Development Model (SDM) [22]. SDM uses ideas from social control, social learning and differential association theory (which postulates that the skills, attitudes and values relating to anti-social behaviour are learned through interaction with others) [23,24] and allows for the changing weight of social influences through the life course. For example, whilst the principal influence on very young children would typically be the family, peers also become important in shaping older children's behaviour. Where children perceive limited closeness to family members, influence from peers may become greater than that of family members [25]. SDM hypothesises that social behaviour is learned through interactions with others, resulting in formation of attachments which may have a lasting effect on behaviour, through supporting development of skills, norms and values. Attachment to others who offer opportunities for and reward prosocial behaviour protects against antisocial behaviour, whilst attachment to those who provide opportunities and rewards for antisocial behaviour is a key risk factor [23,24,26].

One key protective factor commonly studied in relation to children's substance misuse is parental monitoring. Parental monitoring is usually defined as parents' knowledge of the whereabouts and associates of their children, and is related to rule-setting (e.g. about what time young people should return home) [14]. SDM categorises monitoring as a type of "external constraint" on young people's behaviour [24]. Parental monitoring has been shown to protect against adolescent alcohol misuse [27], and may be a necessary but not sufficient condition for influencing children's behaviour [14]. However, rather than a one-way process, Dishion and McMahon see parental monitoring as integral to an ongoing positive relationship between parent and child and as closely linked to the quality of parent-child relationships [28]. Indeed, some Swedish research suggests that 'monitoring' is a misnomer, with the most important factor increasing parents' awareness of their children's social activities being disclosure by children. Rather than a product solely of parental surveillance, disclosure emerges from a reciprocal process between parents and children [29]. Research with Mexican families found that more time spent with the family resulted in more parental monitoring, supporting the view that effective monitoring is a product of other family interactions.

Rule-setting and monitoring which does not arise out of close interaction may have the opposite effect from that intended. Indeed, one recent intervention which encouraged parents to set clearer rules and actively monitor children's activities led to increased family conflict, perhaps due to a focus upon surveillance in isolation from other family interactions [30]. It is likely that the specific focus on discouraging substance misuse led to introduction of rule setting and monitoring which was incongruent with usual styles of parenting. Associations of parental monitoring with children's drinking behaviour therefore cannot be understood in isolation and analyses of its impacts clearly need to take into account related risk and protective factors.

Family conflict and closeness are respectively key risk and protective factors which have been shown to be associated with substance misuse and other antisocial behaviour [31-33]. Family conflict has been linked to adolescent alcohol misuse either directly [34], or through reducing effectiveness of parental monitoring [35]. Parental attitudes favouring antisocial behaviour are known to increase the risk that children will behave antisocially [15,36], likely including alcohol misuse given that the latter is closely linked to other kinds of antisocial behaviour [37]. Indeed, adolescents' misuse of alcohol and other substances has been found to be strongly related to perceptions of parental attitudes towards substance misuse [38]. Some studies have suggested that parents' non-permissive attitude towards drugs may be more influential than their own misuse of drugs [39], although one found that actual behaviour and family structure were better predictors of adolescent alcohol misuse [40]. Having a family member who misuses alcohol is a risk factor for children themselves misusing alcohol or other substances [41,42], whilst having siblings who misuse alcohol or engage in other antisocial behaviour is a particularly strong risk factor [43-45]. One study suggests that rather than direct modelling however, impacts of substance misuse amongst family members occur due to the higher levels of conflict and poorer parent-child relationships in families where one or more members misuse alcohol or other substances [46].

The aims of this study are to examine children's perceptions of protective (parental monitoring, family closeness) and risk factors (conflict, liberality of parental attitudes towards antisocial behaviours and family history of substance misuse) within their family environment, the extent to which these factors are associated with one another, and the associations of these risk and protective factors with children's self reported drinking behaviour. The study was commissioned by a city council's Children and Young People Strategy Unit, who aimed to use a dataset collected from a survey of twelve local schools as a basis for informing practice and policymaking in the field of alcohol misuse prevention. To our knowledge, the study is the first large scale analysis of relationships between these factors and children's drinking behaviours using data from a population in Wales, and is one of a relatively small number of UK studies contributing to the much larger body of research based mainly in the United States.

## Methods

### Background

The survey was conducted by Communities that Care (CTC), a community development organisation focusing upon risk and preventative factors in young people's social environments for a range of behavioural and well-being outcomes [47]. The study involved secondary data analysis of a de-identified cross-sectional dataset provided by CTC, collected in schools in 2008. The study was exempted from ethical review given that data were fully deidentified [48].

### Sampling

All 16 schools within a single Urban district in Wales, UK were invited to participate. The survey was completed by a total of 6,628 pupils in years 7 to 11 (i.e. aged 11-16 years) attending 12 state-maintained secondary schools.

### Data collection procedures

Standardised guidelines for administration of the CTC Youth Survey are reported elsewhere [49]. In brief, teachers were provided with questionnaires to administer to children during school times. Schools were provided with letters to send home to parents to make them aware of the survey, and teachers highlighted to children that participation was voluntary, offering children the opportunity to opt out. Teachers were asked to administer the questionnaires under examination conditions to minimise conferring. Sealable envelopes were provided for children to place their completed and anonymised questionnaires in, before these were returned to researchers.

### Measures

#### Children's alcohol consumption behaviours

Children were asked several questions about alcohol consumption behaviours which formed dependent variables for this study. The first was simply 'Have you ever had more than a sip or two of an alcoholic drink?' requiring a yes or no response. Children who reported trying alcohol were then asked 'How many times have you drunk alcohol in the last 4 weeks?' and 'In the last four weeks, how many times have you had five or more alcoholic drinks in a row?', with response options of never, 1-2, 3-5, 6-9, 10-19 or 20 or more. Children who reported trying alcohol were asked to indicate whether they were frequent drinkers (i.e. 'Do you drink alcohol frequently, that is, at least once a week?'), and whether they had ever been seriously drunk.

#### Family functioning

The questionnaire contained 18 items relating to family functioning, all measured on a 4 point likert-scale (labelled 'NO!', 'no', 'yes', 'YES!') to indicate varying levels of agreement with each statement. A rotated factor solution from factor analysis of these 18 items presented in Table 1 indicates 4 distinct factors emerging from these items. Factors were labelled 'parental monitoring', 'family closeness', 'family conflict' and 'family violence'. The first two factors were considered protective factors, whilst family conflict and violence were considered risk factors. Factor loadings of less than 0.5 are suppressed (i.e. automatically deleted) to aid readability. All four factors demonstrated acceptable internal consistency, with Cronbach's alpha coefficients of 0.80 for parental monitoring, 0.79 for family closeness, 0.77 for family conflict and 0.82 for family violence.

**Table 1:** Rotated factor solution* from factor analysis of 18 items relating to family functioning

	Factor 1 (Parental monitoring)	Factor 2 (Family closeness)	Factor 3 (Family conflict)	Factor 4 (Family violence)
My family has clear rules about alcohol and using drugs	.61			
If I drank some alcohol without my parents' permission, I would be caught by my parents	.67			
If I played truant from school, I would be caught by my parents	.61			
The rules in my family are clear	.66			
My parents want me to phone if I'm going to be late getting home	.59			
My parents ask me regularly if I've done my homework	.54			
When I'm not at home, one of my parents knows where I am and who I am with	.61			
My parents would know if I didn't come home on time	.61			

My parents give me lots of chances to do fun things with them		.70		
My parents ask me what I think before family decisions affecting me are made		.66		
If I had a personal problem, I could ask my parents for help		.63		
How often do your parents tell, or show you that they are proud of you?		.80		
How often do your parents notice when you are doing something well?		.79		

People in my family often insult or yell at each other			.82	
People in my family have serious arguments			.78	
We argue about the same things in my family over and over again			.80	

Adults in my home sometimes try to hurt me, for example by kicking, hitting or pushing me				.84
Adults in my home sometimes try to hurt each other, for example by kicking, hitting or pushing each other				.85

*factor loadings <0.50 are suppressed (i.e. automatically deleted from the table)

#### Children's perceptions of parental attitudes towards 'deviant' behaviours

The questionnaire contained 9 items on perceptions of parental beliefs regarding how wrong it would be for them (the child) to engage in a range of behaviours, all measured on a 4 point likert-scale (from 'very wrong' to 'not wrong at all'). The rotated factor solution presented in Table 2 illustrates the two factors emerging from these items. These factors were labelled i) parental attitudes towards alcohol and petty crime, and ii) parental attitudes towards substance misuse. The item regarding under-age pregnancy did not load onto either factor and was dropped from analysis. Both factors demonstrated acceptable internal consistency (Parental attitudes to alcohol and petty crime α = .78; Parental attitudes to substance misuse α = .74).

**Table 2:** Rotated factor solution* including items relating to perceptions of parental attitudes towards 'deviant' behaviours

How wrong do your parents feel it would be for you to:	Factor 1 (Parental attitudes to alcohol and petty crime)	Factor 2 (Parental attitudes to substance use)
Steal something	.69	
Pick a fight with someone	.78	
Draw graffiti on buildings without permission	.72	
Drink alcohol regularly	.64	
Play truant from school	.62	

Smoke cigarettes		.60
Smoke cannabis		.88
Use drugs like ecstasy. LSD or cocaine		.89

Become pregnant, or get someone pregnant**		

*factor loadings <0.50 are suppressed (i.e. automatically deleted from the table)

**item dropped due to loading on neither factor

#### Family history of alcohol or substance misuse

The questionnaire contained two items regarding family history of alcohol or substance misuse. The first asked 'Did any of your brothers or sisters drink alcohol frequently before the age of 18?' Three response options were available; yes, no, or 'I don't have any brothers or sisters'. As the present study was interested in whether or not children reported having siblings who modelled alcohol misuse before the age of 18, this was converted into a dichotomous variable, comparing those who said yes (i.e. did have siblings who modelled alcohol misuse behaviours) with those who gave another response (i.e. did not have siblings or had siblings who did not model alcohol misuse behaviours). The second item was 'Has any member of your family ever had a serious substance misuse problem?' with response options of yes or no.

#### Age of first trying alcohol

Children were asked to indicate how old they were when they first tried alcohol. Response options were 10 or younger, 11, 12, 13, 14, 15 or 16 years.

#### Demographic covariates

Children were asked to indicate their year group and gender on the questionnaire. These details were used as covariates in multivariate analyses, with year group used as a proxy for age.

### Analysis

Summated scales were constructed to represent the independent variables identified by factor analyses above, through summing values for all items relating to each scale and dividing by the number of items. In order to minimise data loss, where items were missing, these were imputed with the mean value for all remaining items. Data were excluded if less than half of items for a scale were completed.

In order to explore interrelatedness of ordinal independent variables, these were correlated using Spearman's Rank Correlation. This was favoured over Pearson's Product Moment correlation due to the skewed nature of all family functioning variables, with risk factors positively skewed whilst protective factors were negatively skewed. Associations of ordinal independent variables with binary independent variables (family history items) were assessed using Mann Whitney U-tests.

Dependent variables were the binary or ordinal items relating to children's drinking behaviours. Univariable associations of all independent variables with binary dependent variables, (i.e. 'Have you ever had more than a sip or two of an alcoholic drink?' and 'Do you drink alcohol frequently, that is, at least once a week?') were assessed using univariable binary logistic regression. For items forming ordinal dependent variables ('How many times have you drunk alcohol in the last 4 weeks' and 'In the last four weeks, how many times have you had five or more alcoholic drinks in a row?), due to small numbers in the higher ends of the distributions, these were condensed into three category ordinal items (i.e. never, 1-2 times or 3+ times). Due to violation of the proportional odds assumption, ordinal dependent variables were examined using multinomial logistic regression, with the largest group ('never') set as the base category. Subsequent multivariable models were constructed in two stages. First, protective factors were entered (i.e. parental monitoring and family closeness), before risk factors were entered alongside significant protective factors. Multivariate models adjusted for age, sex and age of first drinking alcohol. Although the data sample was hierarchical, clustering at the school level could not be accounted for in these analyses, due to removal of school IDs by the owners of the dataset.

## Results

### Response rates

Twelve out of 16 (75%) schools agreed to take part, although 2 excluded year 7 children, and 2 excluded years 7 and 8, considering questions inappropriate for younger children. Return rates for the 8 schools including all year groups ranged from 40 to 89% (Mean = 70% SD = 19%). For the two schools excluding years 7 and 8, return rates were 36% and 42%. For the two schools excluding year 7 children only, return rates were 49% and 63%. The mean response rate for all 12 schools was 64% (SD = 19%). Whilst 6,628 children provided data, analyses focused upon children within school years 7 to 11 (i.e. aged 11-16 years), leading to the exclusion of 117 children who reported either being in year 6, year 12, or who provided no year group details. Hence, 6511 children who provided data were eligible for inclusion, of whom 6125 responded to a question on whether they had tried alcohol. Many analyses however focused specifically on a subsample of 4634 children who reported having tried alcohol.

For the construction of regression models, the dataset was limited to children who provided responses for all independent variables and the dependent variable in question. Chi-squared analyses comparing children providing complete or missing data for regression models in relation to the first dependent variable (i.e. 'trying alcohol') indicated that complete data were slightly less likely to be available for boys than girls (75.3% vs 79.7%: χ2 = 17.3, p < 0.001), and for children in the younger 3 age groups compared to the older 2 (74.9% vs 77.3% χ2 = 4.7, p < 0.05).

### Sample description

Among the whole sample, 3225 (50.7%) children were male. Amongst the subsample of children who reported having tried alcohol, 2223 (49.2%) were male. The whole sample contained 1196 (18.4%) Year 7 pupils, 1164 (17.9%) Year 8 pupils, 1472 (22.6%) Year 9 pupils, 1432 (22.0%) Year 10 pupils and 1248 (19.2) Year 11 pupils. Of children who had tried alcohol, 562 (12.1%) were in Year 7, 723 (15.6%) were in Year 8, 1083 (23.4%) were in Year 9, 1193 (25.7%) were in Year 10 and 1073 (23.2%) were in Year 11.

### Drinking behaviours of 11-16 year olds

As demonstrated in Table 3, approximately three-quarters of children reported having tried alcohol. Of these children, most (65.9%) first tried alcohol aged 12 or younger. Most children who had tried alcohol (66.9%) reported having drunk alcohol at least once in the past 4 weeks, though for most of these children, this was only once or twice. In total, 28.2% of children reported drinking alcohol 3 or more times in the past 4 weeks. Most who had tried alcohol reported that they had not binge drunk in the past four weeks (62.7%). Of the remaining children, 22.9% had binge drunk once or twice in this time period, and the remaining 14.3% had binge drunk 3 or more times. Of children who had tried alcohol, 18.8% classed themselves as frequent drinkers, whilst 39.4% reported that they had been seriously drunk.

**Table 3:** Frequencies of alcohol consumption behaviours amongst secondary school children

		Frequency	Percent
Have you ever had more than a sip or two of an alcoholic drink?	Yes	4634	75.7
	No	1491	24.3

How old were you when you first had more than a sip or two of an alcoholic drink? (only answered if yes to 1)	10 or younger	1054	22.9
	11	1061	23.1
	12	917	19.9
	13	837	18.2
	14	520	11.3
	15	186	4.0
	16	23	.5

How many times have you drunk alcohol in the last four weeks? (only answered if yes to 1)	Never	1508	33.1
	1-2 times	1767	38.8
	3-5 times	819	18.0
	6-9 times	267	5.9
	10-19 times	108	2.4
	20 or more times	85	1.9

In the last four weeks, how many times have you had five or more alcoholic drinks in a row? (only answered if yes to 1)	Never	2847	62.7
	1-2 times	1042	22.9
	3-5 times	423	9.3
	6-9 times	134	2.9
	10-19 times	46	1.0
	20 or more times	52	1.1

Do you drink alcohol frequently, that is, at least once a week? (only answered if yes to 1)	Yes	845	18.8
	No	3656	81.2

Have you ever been seriously drunk? (only answered if yes to 1)	Yes	1739	39.4
	No	2677	60.6

### Associations between independent variables

As demonstrated in Table 4, there was a high degree of interrelationship among the different components of family functioning, with 'protective factors' (i.e. family closeness, parental monitoring) positively correlated with one another, and negatively correlated with 'risk factors' (i.e. family conflict and violence, and liberality of attitudes towards alcohol/petty crime and substance use). For binary independent variables, Mann-Whitney U tests, indicated that children who reported having siblings who drank regularly before the age of 18 reported significantly lower levels of parental monitoring (z = -19.4) and family closeness (z = -13.3), higher levels of family conflict (z = -12.7) and violence (z = -7.5), and more liberal parental attitudes to alcohol and petty crime (z = -14.7) and substance misuse (z = -14.6). Similarly, children reporting having a family member with a substance misuse problem reported significantly lower levels of parental monitoring (z = -11.6) and family closeness (z = -10.7), higher levels of family conflict (z = -13.1) and violence (z = -13.0) and more liberal parental attitudes to alcohol and petty crime (z = -13.0) and substance misuse (z = -11.4). P-values for all tests of difference were below 0.01.

**Table 4:** Spearman's rank correlation coefficients for all ordinal variables of interest (n = 4977)

	Parental monitoring	Family conflict	Family violence	Family closeness	Parent attitudes to substance misuse	Parent attitudes to alcohol and petty crime
Age (school year)	-.34**	.06**	.02	-.23**	.24**	.25**
Parent attitudes to alcohol and petty crime	-.55**	.23**	.19**	-.38**	.42**	
Parent attitudes to substance misuse	-.38**	.16**	.17**	-.25**		
Family closeness	.53**	-.38**	-.32**			
Family violence	-.23**	.39**				
Family conflict	-.27**					

*sig at 5%, ** sig at 1%

### Associations between independent variables and children's self reported drinking behaviour

In univariable analyses (see Table 5), both protective factors (parental monitoring and family closeness) were negatively associated with drinking behaviours, so that as either factor increased, all markers of children's drinking behaviour became less likely. The inverse was observed for family conflict and family violence, with an increase in either of these variables associated with increases in the likelihood of all markers of drinking behaviour. As parental attitudes towards substance misuse and towards alcohol and petty crime became more liberal, drinking behaviours became significantly more likely. Significant increases in likelihood of all markers of drinking behaviours were observed for children reporting having a sibling who drank regularly before the age of 18 or a family member with a history of substance misuse problems.

**Table 5:** Odds ratios from logistic regression analyses (binary and multinomial) examining associations of family functioning with children's self-reported alcohol consumption (*p < 0.05, **p < 0.01, ***p < 0.001)

	Ever tried alcohol (n = 4977)	Frequent drinker (n = 3651)	Ever been seriously drunk (n = 3594)	Drunk alcohol in past 4 weeks (n = 3697)		Binge drunk in last 4 weeks (n = 3687)	
				1-2 times	More than twice	1-2 times	More than twice
**Univariable associations**							
Family closeness	0.51*** (0.46 to 0.56)	0.49*** (0.44 to 0.54)	0.54*** (0.49 to 0.59)	0.73*** (0.65 to 0.82)	0.48*** (0.43 to 0.54)	0.60*** (0.54 to 0.67)	0.45*** (0.40 to 0.51)
Parental monitoring	0.16*** (0.14 0.19)	0.19*** (0.16 to 0.23)	0.22*** (0.19 to 0.25)	0.32*** (0.27 to 0.38)	0.11*** (0.09 to 0.13)	0.24*** (0.20 to 0.28)	0.11*** (0.09 to 0.13)
Family conflict	1.63*** (1.50 to 1.78)	1.42*** (1.29 to 1.57)	1.38*** (1.27 to 1.50)	1.28** (1.16 to 1.40)	1.51*** (1.36 to 1.67)	1.24*** (1.12 to 1.36)	1.40*** (1.25 to 1.57)
Family violence	1.46*** (1.26 to 1.69)	1.70*** (1.49 to 1.94)	1.42*** (1.26 to 1.60)	1.20* (1.03 to 1.40)	1.57*** (1.37 to 1.85)	1.34*** (1.16 to 1.55)	1.77*** (1.53 to 2.06)
Parental attitudes to substance misuse	4.68*** (3.35 to 6.54)	4.47*** (3.70 to 5.41)	4.12*** (3.34 to 5.07)	2.83*** (2.06 to 3.90)	8.30*** (6.06 to 11.35)	5.92*** (4.58 to 7.64)	10.85*** (8.32 to 14.14)
Parental attitudes - alcohol petty crime	4.50*** (3.77 to 5.38)	3.60*** (3.08 to 4.20)	2.98*** (2.60 to 3.42)	2.21*** (1.86 to 2.62)	4.80*** (4.00 to 5.76)	3.01*** (2.56 to 3.53)	5.41*** (4.50 to 6.50)
Brothers or sisters drank frequently	5.59*** (4.56 to 6.85)	2.94*** (2.48 to 3.47)	2.64*** (2.30 to 3.04)	1.89*** (1.60 to 2.24)	3.78*** (3.16 to 4.51)	2.76*** (2.35 to 3.24)	3.31*** (2.74 to 3.01)
Family member substance problem	3.15*** (2.48 to 4.00)	2.43*** (2.01 to 2.93)	2.61*** (2.21 to 3.10)	1.32* (1.07 to 1.62)	2.21*** (1.79 to 2.73)	1.81*** (1.51 to 2.24)	2.77*** (2.23 to 3.43)

**Multivariate analysis - Protective factors**							
Family closeness	0.97 (0.86 to 1.09)	0.86* (0.75 to 0.98)	0.91 (0.81 to 1.03)	1.08 (0.95 to 1.22)	1.06 (0.91 to 1.22)	0.98 (0.86 to 1.12)	0.97 (0.82 to 1.14)
Parental monitoring	0.23*** (0.19 to 0.28)	0.27*** (0.22 to 0.33)	0.31*** (0.26 to 0.37)	0.35*** (0.28 to 0.43)	0.16*** (0.12 to 0.20)	0.29*** (0.24 to 0.36)	0.15*** (0.12 to 0.19)

**Multivariate analysis - Parental monitoring and risk factors**							
Parental monitoring	0.38*** (0.31 to 0.46)	0.41*** (0.33 to 0.52)	0.41*** (0.34 to 0.50)	0.45*** (0.36 to 0.55)	0.25*** (0.20 to 0.32)	0.43*** (0.35 to 0.53)	0.26*** (0.20 to 0.34)
Family conflict	1.27*** (1.14 to 1.41)	0.92 (0.79 to 1.07)	1.04 (0.94 to 1.16)	1.14* (1.02 to 1.27)	1.07 (0.94 to 1.22)	0.93 (0.82 to 1.05)	0.84* (0.72 to 0.98)
Family violence	.90 (0.76 to 1.07)	1.12 (0.94 to 1.31)	0.97 (0.82 to 1.13)	0.93 (0.78 to 1.11)	0.94 (0.77 to 1.15)	1.02 (0.83 to 1.22)	1.14 (0.92 to 1.40)
Parental attitudes to substance misuse	0.79 (0.58 to 1.08)	1.94*** (1.56 to 2.42)	1.52*** (1.20 to 1.91)	1.20 (0.86 to 1.68)	2.15*** (1.54 to 3.01)	2.26*** (1.70 to 2.99)	3.05*** (2.27 to 4.11)
Parental attitudes - alcohol petty crime	1.67*** (1.34 to 2.09)	1.30* (1.05 to 1.61)	1.27** (1.05 to 1.54)	1.27** (1.03 to 1.57)	1.30** (1.02 to 1.65)	1.44** (1.17 to 1.77)	1.54** (1.20 to 1.99)
Brothers or sisters drank frequently	3.14*** (2.53 to 3.91)	1.87** (1.55 to 2.26)	1.69*** (1.44 to 1.98)	1.49*** (1.24 to 1.78)	2.32** (1.84 to 2.76)	1.97*** (1.65 to 2.36)	2.02*** (1.61 to 2.53)
Family member with substance problem	1.72*** (1.32 to 2.24)	1.33*** (1.06 to 1.67)	1.73*** (1.43 to 2.14)	0.97 (0.77 to 1.22)	1.06 (0.82 to 1.37)	1.25 (0.99 to 1.56)	1.43** (1.10 to 1.88)
Nagelkerke R^2^	0.29	0.26	0.31	0.26		0.32	

Multivariate models were constructed in two stages; first considering protective factors and then risk factors. Entry of parental monitoring and family closeness into a single model in step 1 led to associations of family closeness with children's self-reported drinking behaviours becoming non-significant in all cases with the exception of a marginal association with self classification as a frequent drinker, suggesting that univariable association of family closeness with self-reported drinking behaviours were explained by the strong relationship between family closeness and parental monitoring (see Table 4). Step 2 therefore excluded family closeness, and combined parental monitoring with hypothesised risk factors. After entry of risk factors, parental monitoring remained consistently and inversely associated with all markers of drinking behaviour. Whilst family conflict remained a significant predictor of whether the child had tried alcohol, remaining associations with drinking behaviour became non-significant. All associations of family violence with consumption behaviours became non-significant. Hence, family conflict and violence, whilst associated with drinking behaviours in univariable analyses, did not appear to be consistent independent predictors after accounting for other factors within the model.

Parental attitudes to substance misuse remained a significant predictor of all alcohol consumption behaviours, with the exception of whether the child had tried alcohol. Parental attitudes to alcohol and petty crime remained significantly associated with all markers of drinking behaviour, with the likelihood of all drinking behaviours increasing as the child reported more liberal parental attitudes. Having a sibling who drank regularly before the age of 18 remained significantly associated with increased likelihood of all markers of alcohol consumption, whilst having a family member with a history of substance misuse problems remained a significant predictor of all alcohol consumption markers, with the exception of frequency of alcohol consumption in the past 4 weeks.

## Discussion

The results of the present study are broadly consistent with evidence that while the overall prevalence of alcohol consumption among young people has fallen in recent years, the volume of alcohol consumed by some individuals has increased, with a trend towards greater alcohol consumption by younger adolescents [7]. Self-reported alcohol misuse in this survey suggests that weekly drinking was marginally less prevalent than in Wales as a whole, whilst the incidence of drunkenness was comparable. Nearly 19% of pupils aged 11-16 in this study reported drinking weekly compared with averages of 21% (girls) and 24% (boys) of 11-15-year-olds completing the HBSC survey. In the HBSC survey, 28% of girls and 29% of boys reported having been drunk at least twice [8]. In the present study, 39% of pupils who had tried alcohol (approximately 29% of all pupils) reported that they had been seriously drunk. This comparison should perhaps be treated with caution given that the exclusion of younger pupils in four of the twelve schools means that the CTC sample includes a larger proportion of older pupils than the HBSC sample. Nevertheless, it is clear that the survey area is not exempt from the more general concerns about the large number of young people who misuse alcohol.

Previous research has stressed the importance of effective timing of prevention activities, both in relation to development of family relationships, and young people's drinking practices [20]. The present research found that a significant proportion of young people started drinking alcohol by age 10, and most (65.9%) had first tried alcohol aged 12 or younger. Gruber, et al. found that young people aged 10-12 who drank were particularly vulnerable to alcohol dependency, and that delaying initiation by a year might have significant impacts in reducing alcohol misuse over the longer term [18]. Hence, substance misuse prevention programmes might usefully be offered whilst children are still at primary school before alcohol consumption (or regular consumption) starts. Our findings also highlight the importance of developmentally appropriate support for parents with adolescent children, in order to maximise protective factors linked to family functioning. While the CTC Youth Survey has previously been used in the UK to measure both young people's alcohol-related behaviours and pertinent risk and protective factors (such as the survey conducted in Plymouth, England) [50] the current study is the first to analyse the relationships between these aspects of the survey within a UK context.

Evidence from overseas studies indicating that increased parental monitoring is linked to a decrease in young people's alcohol misuse [19,31,35,51,52] was supported by findings in this study. The role played by formal rule-setting and monitoring is ambiguous but seems likely to be only one element within an array of family interactions influencing children's alcohol use. Interestingly, a recent Hungarian study which examined protective factors for binge drinking among high school pupils found that although parental monitoring and acceptance of parents' values by adolescents (filial piety) were important, social support by parents and joint activities within families (such as eating dinner together) did not remain significant factors in final regression models [53]. The authors suggest that this may have been due both to cultural context (given that social support and joint activities were found to be protective factors in previous US studies), and the changing nature of parent-child relationships during adolescence.

Similarly, in the present study, family closeness was not a significant predictor after entry of parental monitoring. However, in the present study, parental monitoring and rule setting around alcohol was inseparable from more general communications regarding the child's behaviours, and was shown to be substantially higher amongst children who perceived a high degree of family closeness. Whilst not explicitly framed as a mediational analysis, the conditions for supporting a hypothesis of mediation according to Barron and Kenny [54] were all met in the present study. That is, i) the independent variable (family closeness) was associated with the outcome variables (self-reported drinking), ii) the independent variable was associated with the mediator variable (parental monitoring), iii) the mediator variable was associated with the outcome variables after controlling for the independent variable and iv) the association of the independent variable was eliminated by entry of the mediator variable. Hence, parental monitoring was perhaps an outcome of family closeness, which in turn predicted children's drinking behaviours. Such an interpretation is consistent with Stattin and Kerr's hypothesis that parental 'monitoring' may be linked to more open communication by children within close families [29].

Higher levels of parental monitoring were also associated with lower levels of violence and conflict, reduced likelihood of family history of substance misuse and less liberal attitudes towards alcohol and petty crime. Hence, parental monitoring appeared a practice typical to parents who modelled less antisocial behaviours and perhaps provided less opportunity for such behaviours. Whilst parental monitoring appears important, some research indicates that changing monitoring without considering the nature of family interactions which give rise to closer monitoring may be counterproductive [30]. Findings highlight the need for further exploration of the influence of family and wider cultural contexts on parental knowledge and regulation of children's behaviour. Parental monitoring remained a significant predictor of alcohol consumption after adjusting for all of these factors. Whilst beyond the remit of the present study, interactions between risk and protective factors, and the multiplicative impact of limited parental monitoring alongside higher levels of risk factors deserve closer scrutiny in future research.

A relationship was found here and elsewhere [9,38,55] between increased alcohol misuse reported by young people and perceptions of more liberal parental attitudes towards consumption of alcohol and other substances and towards petty crime. Univariable models showed significant associations between family conflict and violence and children's drinking behaviour. However in multivariate models, associations of conflict and violence became non-significant. Hence, the relationship of family conflict and violence with young people's alcohol use may be confounded or possibly mediated, by other factors, rather than having the direct effect identified in other research [34]. Examination of the pathways through which family conflict and violence may impact children's drinking behaviours, whilst beyond the scope of this study, is an important direction for future research.

The finding that adolescent alcohol misuse was associated with family history of substance misuse problems was consistent with other research [41] as was the association with having a sibling who drank frequently before the age of 18 [43-45]. However the independence of these factors from other risk and protective factors contrasts somewhat with Kroll's review, which suggests that parental substance misuse influences children through its effects on family interactions [46] or via perceived parental attitudes [42]. The question in the CTC survey does not however ask specifically about parental substance misuse, and answers may refer to a variety of close or distant family members. A high number of responses referring to family members outside the parental home might account for the apparent independence of this influence, because residence elsewhere may have less effect on interactions between members occupying the same family home. This explanation might also account for a similar independent effect found for siblings who drank regularly before the age of eighteen, because data could refer to a brother or sister living in a different household from that of the respondent. The implications of these findings are difficult to estimate without information of the relationship to respondents, and the age and residence of such family members. In addition, given the cross sectional nature of the design, it is possible that other unmeasured aspects of family functioning mediate the impact of family role models.

Using the Social Development Model as a framework explained the main findings well. As previously described, the model proposes that interaction of children and young people with other family members forms the basis of attachments which may lead to adoption of prosocial or antisocial attitudes, beliefs and behaviour. Attachment to others who support prosocial behaviour through for example, close monitoring and communication between parent and child, may promulgate prosocial behaviour and reduce risk of alcohol misuse. Young people who form attachments to others who model behaviours such as substance misuse however, or are liberal in their attitudes towards misuse of alcohol or other substances by the child, will likely be at greater risk of developing more antisocial attitudes, beliefs and behaviour surrounding substance misuse.

Whilst many UK based interventions have been directed through the classroom, attention ought to be paid to supporting parenting behaviours which reduce children's risk of alcohol misuse. As the child grows older and develops social contacts outside the family, friends' approval becomes an important predictor of whether, and how much, the child drinks [37]. Nevertheless, parents' influence may remain much stronger than that of friends into late adolescence, particularly where family relationships are perceived by the child as close [9,55]. Prevention interventions need to address the broad determinants of alcohol consumption rather than focusing narrowly on raising children's awareness or increasing their knowledge about alcohol. Whilst the current study did not seek to make detailed policy recommendations, findings point to the need for a multifaceted approach to alcohol misuse prevention, encompassing school-based education, broad-based parenting and family programmes, treatment of substance misuse within families, and support services for families experiencing domestic violence. Such a range of services are likely to be provided by a range of different agencies, both within and without the substance misuse sector.

A number of future directions for research have been highlighted, including exploring the pathways through which family conflict and violence may predict alcohol consumption and the multiplicative impacts of risk factors. In addition, whilst this study has examined associations of children's perceptions of family functioning, future directions for research include exploring the extent of agreement between children's perceptions of family functioning and the reports of parents themselves, as well as associations of parental reports of parenting behaviours with children's drinking behaviours. Parents' may underestimate their children's drinking behaviour, particularly where communication between parent and child is limited, and hence children's self reports of drinking behaviour may offer greater validity than those of parents [56]. However, parents and young people may have very different perceptions of issues such as what constitutes rules and monitoring, and how strict or clear family rules are.

### Study strengths and limitations

A number of strengths and limitations of the present study merit consideration. The study benefits from a large, representative sample of children within one Welsh city. Whilst using questionnaire items over whose design and administration the authors had no control, thorough factor analyses allowed for construction of independent variables which were both sufficiently related to the research questions and sufficiently distinct from one another to be considered as separate but inter-related constructs. Whilst somewhat strong associations (i.e. r > 0.50) were observed between parental monitoring and two other variables (family closeness and parental attitudes to alcohol and petty crime), exploratory analyses indicated that these were not sufficiently inter-related to cause multicollinearity. Indeed, family closeness was not included in final models, whilst parental monitoring and parental attitudes to alcohol and petty crime remained significant independent predictors despite a large degree of shared variance.

However, it should be conceded firstly that data are cross sectional, and hence cause and effect cannot be demonstrated. Secondly, self report data is likely subject to social desirability biases. Thirdly, statistical analyses were limited by the removal of school-level identifiers from the dataset. This meant that it was not possible to assess or account for violations of the assumption of independence due to the hierarchical nature of the data sample. Cluster effects are related to the degree of intracluster correlation within the data, as well as the size of clusters, increasing as either of these factors becomes larger. Hence, the dataset, rather than including 6,000 independent units of analysis, is comprised of 12 clusters (i.e. schools) with an average size of 500 children per cluster. Given this large cluster size, even a small intra-cluster correlation would lead to large cluster effects. Therefore it is likely that associations are estimated with an artificially high level of certainty. Adjustment for clustering would not change odds ratio estimates, but would likely widen confidence intervals, leading some more borderline trends to become non-significant. Whilst the authors attempted to negotiate the reintegration of anonymised IDs, these requests were declined as the holders of the dataset had informed schools that they would be removed. Finally, somewhat variable response rates were observed across schools, due in large part to certain schools declining access to younger year groups, with implications for external validity. It was not possible to compare responses of children in schools with high or low response rates due to the aforementioned removal of school IDs. Furthermore, whilst efforts were made to minimise data loss by including all children who provided at least half complete responses on each scale, relatively large quantities of missing data were observed with boys and younger children slightly less likely to provide complete data, potentially introducing additional biases. Nevertheless, the study has demonstrated some compelling associations between children's perceptions of their family contexts and their own drinking behaviours.

## Conclusions

This analysis confirms other findings of a strong association between family relationships and young people's alcohol misuse and also supports the hypotheses of the Social Development Model regarding the influence of parent-child interactions on young people's subsequent behaviour. Future programmes to reduce young people's alcohol misuse should target the families of young children, especially with respect to improving communication between parents and children. There is a need to look beyond classroom-based education and to assess what other support is available for families. This implies a requirement for multi-agency work to address a broad spectrum of support needs. Useful directions for further research would include exploration of the quality of family communication, particularly as it relates to explicit rule setting or more subtle regulation of children's behaviour; and how correlates of drinking behaviours are moderated by children's age.

## Competing interests

The authors declare that they have no competing interests.

## Authors' contributions

GM developed research questions, conducted data analysis and drafted sections of the manuscript. HR conducted the literature review and drafted sections of the manuscript. JS developed research questions, added to the literature review and contributed to revisions of manuscript drafts. All authors contributed to revisions of and approved the final manuscript.
